# PPI network analyses of human WD40 protein family systematically reveal their tendency to assemble complexes and facilitate the complex predictions

**DOI:** 10.1186/s12918-018-0567-9

**Published:** 2018-04-24

**Authors:** Xu-Dong Zou, Ke An, Yun-Dong Wu, Zhi-Qiang Ye

**Affiliations:** 1 Lab of Computational Chemistry and Drug Design, Laboratory of Chemical Genomics, Peking University Shenzhen Graduate School, Shenzhen, 518055 People’s Republic of China; 2 College of Chemistry, Peking University, Beijing, 100871 People’s Republic of China

## Abstract

**Background:**

WD40 repeat proteins constitute one of the largest families in eukaryotes, and widely participate in various fundamental cellular processes by interacting with other molecules. Based on individual WD40 proteins, previous work has demonstrated that their structural characteristics should confer great potential of interaction and complex formation, and has speculated that they may serve as hubs in the protein-protein interaction (PPI) network. However, what roles the whole family plays in organizing the PPI network, and whether this information can be utilized in complex prediction remain unclear. To address these issues, quantitative and systematic analyses of WD40 proteins from the perspective of PPI networks are highly required.

**Results:**

In this work, we built two human PPI networks by using data sets with different confidence levels, and studied the network properties of the whole human WD40 protein family systematically. Our analyses have quantitatively confirmed that the human WD40 protein family, as a whole, tends to be hubs with an odds ratio of about 1.8 or greater, and the network decomposition has revealed that they are prone to enrich near the global center of the whole network with a fold change of two in the median *k*-values. By integrating expression profiles, we have further shown that WD40 hub proteins are inclined to be intramodular, which is indicative of complex assembling. Based on this information, we have further predicted 1674 potential WD40-associated complexes by choosing a clique-based method, which is more sensitive than others, and an indirect evaluation by co-expression scores has demonstrated its reliability.

**Conclusions:**

At the systems level but not sporadic examples’ level, this work has provided rich knowledge for better understanding WD40 proteins’ roles in organizing the PPI network. These findings and predicted complexes can offer valuable clues for prioritizing candidates for further studies.

**Electronic supplementary material:**

The online version of this article (10.1186/s12918-018-0567-9) contains supplementary material, which is available to authorized users.

## Background

The WD40 repeat proteins constitute one of the largest protein families in eukaryotes [1], and more than 1% of human protein-coding genes encode WD40 proteins [2]. Studies found they participated in signal transduction, transcriptional regulation, protein degradation, cytoskeleton assembly, DNA damage repair, cell cycle regulation, and so forth, leading to an understanding of their involvement in many fundamental cellular processes [1, 3, 4]. The β subunits of heterotrimeric G proteins, as the most well-known WD40 proteins, transduce transmembrane signals mediated by GPCRs [5]. A set of WD40 proteins containing F-box, as key modules in SCF-ubiquitin ligases, recognize substrates and are responsible for their ubiquitin-dependent degradation [6]. Study on their interactions with other molecules is indispensable to understand their functions.

Available crystal structures have shown that WD40 domain exhibits a β-propeller structure exposing its top, bottom, and side surfaces. Through these large surfaces, they interact with other molecules and form complexes to perform their versatile functions [1, 3, 5, 7–10]. For instance, FBXW7 utilizes its top surface to interact with the substrates [11], and PALB2 interacts with BRCA2 through its side surface [12]. It is reasonable to assume that these structural characteristics can offer great potential of interactions and make them as scaffolds for complex assembling. Based on this consideration, researchers have speculated that the whole WD40 protein family may act as nodes with high connectivity (i.e., hub) in the protein-protein interaction (PPI) network [1, 4]. However, this inference has not been validated by using PPI networks directly. In addition, what roles the whole WD40 protein family play in organizing PPI networks remain unclear, and need to be elucidated for better understanding of their functions. Another crucial problem is to identify their involvements in functional complexes, but whether the information drawn from the network analyses could be utilized in the prediction of WD40-associated functional complexes is currently unexplored. To address these issues, quantitative and systematic analyses of WD40 proteins, as a whole family, from the perspective of PPI network are highly required.

High-throughput approaches such as yeast two-hybrid (Y2H) and affinity purification-mass spectrometry (AP-MS) have generated large-scale PPI data sets [13–15], and many online databases, such as MINT [16], MIPS [17], HPRD [18], and STRING [19], have integrated comprehensive information of both high-throughput and low-throughput PPIs. The accumulation of PPI data makes it possible to construct PPI networks and to perform systematic studies based on available network analysis methods. These network analyses, focusing on either the static features such as connectivity and location or the dynamic features such as co-expression coefficients, have in fact obtained various achievements [20–25]. All of these have offered the feasibility for a network analysis on WD40 proteins.

In this work, we adopted the human PPI data set from HIPPIE [26, 27] database to build two human PPI networks with different confidence levels. Using these two networks in parallel, we then analyzed the network characteristics of human WD40 proteins, including their centrality measures such as degree, the location (*k*-value in *k*-core decomposition), and the co-expression correlation coefficient between a node and its interacting partners, to help understand their roles in organizing the PPI network. Finally, we predicted WD40 protein-associated complexes based on the network topological features, and evaluated its performance. The overall pipeline of this work is illustrated in a flowchart (see Additional file 1: Figure S1).

## Results

### Two PPI networks with different confidence levels

We curated two human PPI data sets with different confidence levels from HIPPIE database [26, 27]. One contains all the interactions from HIPPIE after data cleaning (namely ALL-PPI), while the other only consists of the PPIs with high confidence scores (namely HC-PPI, see Methods). In brief, ALL-PPI contains 229,137 interactions among 16,226 human proteins, while HC-PPI contains 66,789 interactions among 11,108 human proteins, accounting for about 29% of ALL-PPI (Table 1, see Additional file 2: Table S1 and Additional file 1: Figure S2). The network analyses were performed on HC-PPI and ALL-PPI in parallel, which ensured that we could obtain robust and consistent conclusions. This was also helpful to the evaluation of the impact on the inferences stemming from PPIs with different confidence levels.

**Table 1:** Basic information of the ALL-PPI and HC-PPI networks

	HC-PPI	ALL-PPI
Proteins (nodes)	11,108	16,226
Interactions (edges)	66,789	229,137
WD40 proteins	203	242
Components	76	8
Occupation of main component	96%	99%

There are 242 and 203 WD40 proteins in ALL-PPI and HC-PPI, respectively, and all of them are located in the main components in the constructed networks. As the main components occupy the majority of the nodes (see Table 1 and more detailed information in Additional file 2: Table S1), further network analyses have been carried out on them only. We have observed that the degrees approximate the power law distribution in both networks (Additional file 1: Figure S3), which is consistent with the well-established opinion that most biological networks follow a scale-free topology [28].

### WD40 proteins tend to be hubs in human PPI networks

For the whole human WD40 protein family, we directly evaluated their tendency of acting as hubs in the PPI networks. There are 123 WD40 hub proteins (with degree greater than 5, see definition in Methods) in HC-PPI network (Table 2). By considering the numbers of hubs and non-hubs in non-WD40 proteins, we have obtained an odds ratio (OR) of 1.82 (*p* = 3.844e-5 in a χ^2^ test, see Methods). The quantitative measure of the odds ratio, which is significantly greater than 1, supports the inference that the whole WD40 protein family indeed tend to act as hubs in HC-PPI network. We performed the same analysis on ALL-PPI network, and the result backs the above inference more strongly (OR = 2.83, *p* = 2.077e-9, see Additional file 1: Table S2). To be more stringent, we also attempted these analyses by using alternative hub definitions with different cutoffs (degree greater than 10 or 15, see Methods), and all confirmed that the WD40 family tend to be hubs (Additional file 1: Table S3). The observation that the OR value in ALL-PPI is much larger than in HC-PPI for each hub definition, indicates that the tendency of WD40s to be hubs may be underestimated when using high confidence PPIs only. Nevertheless, this tendency is significantly larger than that of non-WD40s in all scenarios, demonstrating that our inference is robust.

**Table 2:** Number of hubs and non-hubs of both WD40 and non-WD40 proteins in HC-PPI network

	WD40	Non-WD40	Total
Hub	123	4995	5118
Non-hub	80	5910	5990
Total	203	10,905	11,108

The definition of hub protein is controversial currently. To avoid this, we further compared their degrees directly. In HC-PPI network, the median degree of WD40 proteins is significantly greater than that of non-WD40s (9 vs. 5, fold change ~ 2, *p* = 2.19e-8, Mann-Whitney U test, see Additional file 1: Table S4), which again demonstrates that they possess higher preference of interacting with other proteins than non-WD40s do. Similar results were observed from the analysis on ALL-PPI (24 vs. 11, fold change ~ 2, see Additional file 1: Table S4). Based on the investigations of certain individual WD40s and their structural features, previous studies have speculated that the whole WD40 family may tend to participate frequently in molecular interactions [1, 3, 29]. In this work, directly analyzing the whole set of human WD40 family in the PPI networks has confirmed this inference systematically.

In addition, our analysis has provided quantitative degrees for each WD40 protein, which could be utilized to select candidates for in-depth studies. It is well accepted that proteins with high degree in the network are often associated with important functions [20]. In HC-PPI, the top three WD40 hubs are FBW1A, FBW1B, and DDB1, whose degrees are 108, 102, and 81, respectively (Table 3). FBW1A and FBW1B, which are paralogous to each other, serve as subunits of SCF E3 ubiquitin ligases, and many studies have shown that these two genes regulate cell cycle by degrading related proteins such as Cdc25A and Wee1 [30, 31]. As a linker in DDB1-CUL4-ROC1 E3 ligase, DDB1 was predicted to interact with about 90 other WD40 proteins by sequence similarity search [32]. By degrading corresponding protein substrates, it regulates many fundamental cellular processes, including DNA repair, cell cycle, and DNA replication [33].

**Table 3:** WD40 proteins with high- and low-degrees in both HC-PPI and ALL-PPI network

	Protein	Degree in HC-PPI	Degree in ALL-PPI	Expression
High degree	FBW1A	108	341	High in many tissues
	FBW1B	102	375	High in many tissues
	DDB1	81	240	High in all tissues
Low degree	DC121	1	1	Testis-specific
	EMAL5	1	2	Ovary-preferential
	TBL1Y	1	2	Prostate-specific

Although the whole family tends to be hubs, many individual proteins in this family have very low degrees, and are worth exploring as well. The 3 WD40 proteins with lowest degrees in both HC-PPI and ALL-PPI network are listed in Table 3. According to the database search in PubMed and UniProt [34], they have not been studied widely and their functional annotations remain limited. Interestingly, we found that they show a tissue-specific or tissue-preferential expression pattern (for definition of expression patterns, see Methods) [2, 35]. On the contrary, the three genes with top degrees are prone to express widely (Table 3). Although further confirmation of this correlation is needed, we can speculate that the widely-expressed proteins may interact with different partners in different tissues, and that combining all interactions from different tissues into the overall PPI network has resulted in the high degrees.

Degree is the most simple and intuitive characteristics that describes the centrality of a node. To obtain more comprehensive understanding of their centralities, we also compared other measures including betweenness, closeness, stress, and clustering coefficient, between WD40 and non-WD40 proteins in both HC-PPI and ALL-PPI. All these comparisons have revealed consistent trends (Additional file 1: Table S4), demonstrating the WD40 family indeed tends to have higher centrality levels from multiple perspectives.

### WD40 proteins prefer to locate near the global center of PPI networks

Proteins are hierarchically located in the PPI network, and those with high degrees may be located near the periphery or the center of the whole network [21], which are often referred to as the local center or global center (see Methods for definition), respectively. While the status of a protein to be hub or not provides valuable information for understanding its role in organizing the PPI network, whether a protein tends to be located near the global center or local center can offer additional clues.

**Fig. 1:**
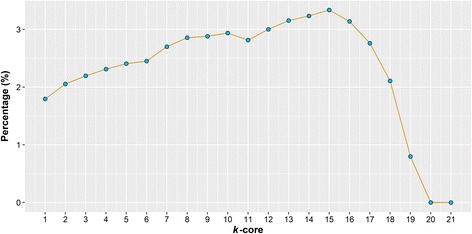
Percentage of WD40 proteins in each *k*-core subnetwork during the decomposition of HC-PPI network. The percentages were obtained by dividing the number of WD40 proteins to that of total proteins in each *k*-core subnetwork

To investigate the distinct locations of WD40 proteins in human PPI network, we performed the *k*-core decomposition (see Methods) for HC-PPI. As shown in Table 4, HC-PPI network can be split into 21 layers, and the WD40 proteins are widely located from layer 1 to 19. The median *k*-value of WD40 proteins is significantly greater than that of non-WD40s (8 vs. 4, fold change = 2, *p* = 8.56e-10, Mann-Whitney U test). As the large *k*-value can indicate the preference of being located near the global center (see Methods), this result has demonstrated that this propensity of WD40 proteins is significantly higher than that of non-WD40s. We further found that the percentage of WD40 proteins in each *k*-core subnetwork increased almost linearly with *k* in a certain range (from 1 to 15 on Fig. 1, linear regression R^2^ = 0.95, *p* = 4.12e-10), further showing that the WD40 protein family is prone to enrich near the global center in a more vivid way. The same analysis was carried out for ALL-PPI network, and similar results were observed (median *k*-value: 20 vs. 10, fold change = 2, see Additional file 1: Figure S4 and Table S5).

**Table 4:** WD40 proteins in different layers by *k*-core decomposition on HC-PPI network

*k*-layer	# of WD40	# of non-WD40	WD40 protein
21	0	26	–
20	0	203	–
19	3	141	MED16, GBLP, COR1C
18	8	130	FBW1A, FBXW7, ARC1B, FBW1B, RBBP4, DDB1, PAAF1, RBBP7
17	11	253	EED, VPRBP, STRN3, WDR5, MEP50, CDC20, STRN, DDB2, WDR1, BUB3, 2ABB
16	12	262	WDR48, WRP73, RFWD2, EIF3B, SEH1, RBBP5, STRN4, PRP19, EIF3I, DCAF7, RAE1L, PRP4
15	10	216	TBL1X, SEC13, GEMI5, NEDD1, SNR40, COPA, FZR, STRAP, COR1B, 2ABD
14	6	216	TBL1R, TLE1, KI21A, TAF5, WDR62, FAN
13	8	277	2ABA, KI21B, TF3C2, 2ABG, LRWD1, LYST, WDR33, WAP53
12	6	281	RPTOR, CAF1B, GBB2, GBB1, WDR82, COR2A
11	7	376	EDC4, WDR61, FBXW5, COPB2, A16L1, WDR20, WDR36
10	13	312	CIR1A, CORO7, APAF, DCA11, PAN2, PLRG1, FBXW4, DTL, TBL3, WDR18, WDR26, PHIP, THOC6
9	12	448	WIPI2, LIS1, NUP37, HPS5, PALB2, WDR90, ERCC8, WDR92, DCAF8, WDR6, NUP43, TAF5L
8	12	426	PI3R4, GBB4, TLE3, WDR83, ELP2, CIAO1, THOC3, WDR35, WDR74, FBXW8, ARC1A, CSTF1
7	7	463	LST8, DC1I2, DCAF4, WDR75, NBEL1, WDR76, HIRA
6	3	551	SMU1, WDR3, DC1I1
5	15	682	SHKB1, DCA10, KCTD3, BRWD1, GBB5, GBB3, AAAS, PWP2, WDHD1, EMAL3, UTP18, PEX7, BOP1, SC31A, WDR24
4	14	802	NBEL2, WDR34, EIF2A, WDR44, STXB5, PRP17, WDTC1, PLAP, TLE2, WDR43, WSB1, FBXW2, UTP15, AHI1
3	17	1078	NLE1, WDR12, TSSC1, WIPI1, DCA13, POC1B, COR1A, IF172, SCAP, AAMP, EMAL4, WDR70, BRWD3, DCAF5, TRAF7, WDR5B, WDR46
2	20	1449	DCA12, FBXW9, WIPI4, EMAL1, LRBA, DMXL2, TEP1, WSB2, EMAL2, WDR59, WDR55, DMXL1, WDR7, PK1IP, PWP1, HERC1, WDR4, TBL2, MIO, U3IP2
1	19	2313	TCPR2, COR2B, WDR47, WDR37, GNB1L, DC121, IFT80, IF122, TBL1Y, SC31B, DC4 L1, MABP1, NOL10, EMAL5, KTNB1, DEND3, WDR81, NBEA, WDR25

It has been demonstrated in the yeast PPI network that proteins located near the global center tend to be essential genes and be conserved in evolution [21]. Hence, we checked three human WD40 proteins with the largest *k*-values in HC-PPI (Table 4). Among them, GBLP (also named RACK1) plays roles in many cellular processes, such as translational repression, PKC signaling pathway, and so forth, and it belongs to the human essential genes reported previously [36]. MED16 is a key component of the Mediator complex which is involved in the transcription regulation of nearly all RNA polymerase II-dependent genes [37], and it is synthetically lethal when knocked out together with MED15 [38]. CORO1C, a member in the Coronin gene family, is associated to many cancers and brain development [39]. In addition, all the three genes are evolutionarily conserved in vertebrates or even in the whole eukaryotes (see Additional file 1: Table S6).

Taken together, the *k*-core decomposition has provided information concerning the WD40 proteins’ locations in the PPI network, which cannot otherwise be derived from the degrees only. These results have shown that WD40 proteins prefer to locate near the global center in organizing the network topology. By identifying WD40 proteins close to the global center, one can further mine the WD40 proteins and prioritize candidates for further investigation.

### WD40 hubs tend to be intramodular hubs

By integrating expression data into the PPI network, previous studies defined two kinds of hubs by the level of co-expression between the hub and its interacting partners (see Methods) [23, 24], where the high level and low level indicate intramodular and intermodular hubs, respectively. These two kinds of hubs display distinctive characteristics consistent with their roles in organizing communications and functions of dynamic protein networks, e.g., the intramodular ones often serve as platforms to assemble complexes [23, 24].

**Fig. 2:**
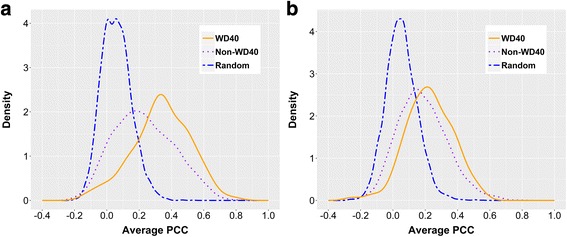
Distributions of average PCCs of WD40 hubs, non-WD40 hubs, and randomized data in HC-PPI network. The solid lines in orange represent the WD40 hubs, the dotted lines in purple denote the non-WD40 hubs, and the longdash lines in blue represent the randomized data. The average PCCs are calculated by using both protein-level expression data (**a**) and RNA-level expression data (**b**)

We measured the co-expression levels between hubs and their partners by calculating the average Pearson correlation coefficients (PCC, see Methods) in the HC-PPI network. As expected, the average PCCs of WD40 and non-WD40 hubs are both higher than those of randomized data (Fig. 2). Furthermore, the average PCCs of WD40 hubs are significantly higher than those of non-WD40 hubs (Fig. 2), indicating that WD40 hubs have higher tendency to be intramodular than non-WD40 hubs. We observed the similar trend for the protein-level (median of average PCCs: 0.343 vs. 0.217 for WD40 and non-WD40 hubs, *p* = 1.7e-10, Mann-Whitney U test) and the RNA-level (0.221 vs. 0.171, *p* = 1.6e-4, Mann-Whitney U test) expression data in the HC-PPI network (see Additional file 1: Table S7). In addition, the difference between WD40 hubs and non-WD40 hubs is evidently larger in protein-level expressions than in RNA-level expressions (Fig. 2 and see Additional file 1: Table S7). As we are studying the interactions at the protein level, the protein-level expressions should be more proper and more direct than RNA-level expressions. Hence, the larger difference observed based on protein-level expression has further strengthened our inference concerning WD40 hubs’ intramodular tendency. The similar analyses were also performed in ALL-PPI network, and led to consistent observations (see Additional file 1: Figure S5 and Table S7).

By using both protein-level and RNA-level expression data, and by using both HC-PPI and ALL-PPI network, these results have provided quantitative clues systematically to support the inference that WD40 hubs, as a whole set, are more prone to being intramodular. This information, in combination with their tendency to be hubs and to locate near the global center, has largely extended our understanding concerning their roles in organizing the PPI network. According to the previous studies on PPI networks, the intramodular hubs tend to assemble complexes [24]. Hence, analyses in this section also directly confirmed previous speculations about their tendency of acting as scaffolds. Taken together, these network analyses may indicate that one can further predict WD40-associated complexes by using the network topology.

### WD40-associated complex predictions

Protein complex, from biological perspectives, represents a group of proteins that interact with each other at the same time and place, forming a multimolecular machine. From a topological perspective, protein complex represents a highly connected subgraph or cluster that has more interactions with each other within it and fewer with the outside of the subgraph [25]. Cliques are such a kind of highly connected subgraphs, where each pair of nodes are linked by an edge, and clique-based methods are useful for predicting complexes from network [40–42]. The previous sections has confirmed at the systems level that WD40 hubs tend to form complexes (Fig. 2), and the clustering coefficients, which measure the trends of nodes to form dense clusters, of WD40 proteins are also much higher than those of non-WD40 proteins (see Additional file 1: Table S4). Therefore, choosing a method simply based on finding cliques may be effective to predict WD40-associated complexes.

By using the HC-PPI network, we detected 1674 maximal cliques (Additional file 3: Table S8). The size of clique ranges from 3 to 16, and many cliques are overlapped with each other. We merged the cliques to obtain a series of predicted complex sets, namely from M05 to M10, according to different levels of the overlap between two cliques (see Methods for the names of the sets). These sets contain from less than 100 to more than 1000 predicted complexes (Additional file 1: Table S9).

**Fig. 3:**
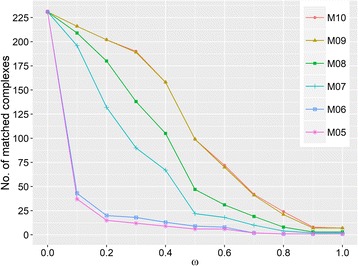
The number of reference complexes matched by the predicted complex sets at different ω scores. Different lines represent different predicted complex sets derived from different merging parameters. The ω at the X-axis denotes the score that determines whether a predicted complex matches a reference one. The Y-axis gives out the number of reference complexes matched by predicted ones at corresponding ω scores

To find out which complex set is the best, we compared them with a reference set containing 234 experimentally-identified human WD40-associated protein complexes extracted from the CORUM database [43] (see Methods and Additional file 4: Table S10). To fulfill these comparisons, we also tried different values of **ω** [44], which was used to determine whether a predicted complex matches one of the reference complexes (Additional file 1: Table S9 and see Methods for the definition of **ω**). As shown in Fig. 3, M10 matches the reference set better than other predicted sets (from M05 to M09) under all ω scores. This result suggests that using the maximal cliques without further merging can effectively predict out true WD40-associated complexes, so the following analyses are all based on M10.

We also tried other well-known methods for comparison, including MCODE [44], ClusterOne [45], and MCL [46]. We found that all the three methods output much less WD40 protein-associated complexes than the clique-based method (Additional file 1: Table S11), indicating that the clique-based method is much more aggressive. Besides, when comparing the predicted complex sets by these three methods to the reference set, we found that the numbers of matched reference complexes are much less than clique-based method under almost all ω scores (Additional file 1: Figure S6), suggesting a higher sensitivity of the clique-based method. In addition to considering the matched number of reference complexes, we further adopted the maximal matching ratio (MMR) to compare these methods [45]. The MMR can measure to what extent the predicted complexes overlap with the matched reference complexes. At the setting of ω > = 0.2, as recommended by MCODE [44], we found that the clique-based method obtained similar or even better MMR (Additional file 1: Table S12), revealing that the clique-based method can detect more true complexes without sacrificing their quality. Taken together, these observations indicated that, although the clique-based method may have a high false positive rate, it can detect much more true WD40 complexes than others.

In order to evaluate the impact on the prediction stemming from PPIs with different confidence, we further performed the clique-based prediction by using ALL-PPI network. It turned out that the M10 matched the reference set best, similar to the case by using HC-PPI network (Additional file 1: Figure S7). As expected, the predicted WD40 protein-associated complexes from ALL-PPI are much more than those from HC-PPI (Additional file 1: Table S13), as the incorporation of many interactions with lower confidence forms more cliques. However, the numbers of matched reference complexes based on ALL-PPI and HC-PPI are similar and are near the total number of reference complexes at the ω > = 0.2 (Additional file 1: Figure S8 and Table S13). This indicates that the clique-based method on ALL-PPI network has output too many positive predictions without contributing much to the true positive predictions, suggesting that clique-based complex prediction by using HC-PPI may be better than by using ALL-PPI.

### Further evaluation of the final predicted complex set

We chose M10 from HC-PPI as the final predicted complex set according to the analysis above, and it matched 202, 190, 158, and 99 known WD40-associated complexes in the reference set at ω scores no less than 0.2, 0.3, 0.4, and 0.5, respectively (see Additional file 1: Table S9). As it is difficult to obtain a suitable negative control data set, it is challenging for us to evaluate the false positive of our prediction directly. However, as protein complexes are groups of proteins that exert functions at the same time and location, it is reasonable to assume that proteins within a true complex have high co-expression relationships. Therefore, we further evaluated the final predicted complex set indirectly by calculating a co-expression score for each potential complex (similar to but different from the average PCC for hub proteins, see Methods).

**Fig. 4:**
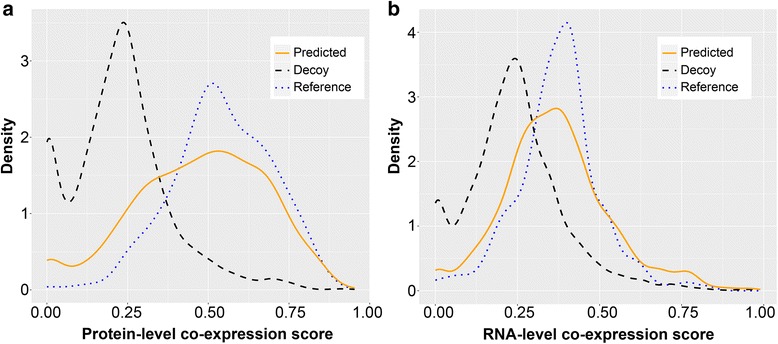
Distributions of the co-expression scores of predicted WD40-associated complexes, reference complexes, and decoy complexes. The orange solid line, the blue dotted line, and the black dashed line represent the distributions of co-expression scores of predicted WD40 complexes, reference complexes, and decoy complexes, respectively. The co-expression scores are calculated by using both the protein-level expression data (**a**) and the RNA-level expression data (**b**)

By comparison (Fig. 4), we observed that both the predicted WD40-associated complexes and the reference complexes presented significantly higher co-expression scores than the decoy complexes, i.e., randomized protein sets (*p* < 2.2e-16 for both tests with protein-level or RNA-level expression data, Mann-Whitney U tests). In addition, the fold changes with the protein-level expression are both larger than 2, and those with the RNA-level expression are both larger than 1.5 (see Additional file 1: Table S14). When the co-expression scores of the predicted complexes were compared to those of reference complexes, the statistically significant difference was observed with protein-level expression data (*p* = 1.015e-6, Mann-Whitney U test), but the fold change of medians is only 1.09 (see Additional file 1: Table S14). With the RNA-level expression data, no statistically significant difference was even observed (*p* = 0.115, Mann-Whitney U tests), and the fold change of medians is only 1.04 (see Additional file 1: Table S14).

The above results provide several indications. First, the co-expression scores are more distinguishable by using the protein-level expression data, which meets our understanding that the protein-level expression data is more suitable for integrating into the PPI network analyses than the indirect RNA-level expression data. Second, the co-expression scores are much higher in the reference complex set than in the decoy complex set, indicating that the co-expression values do have the potential to evaluate the predicted complexes. Third and most important, the much smaller differences between co-expression scores of our predicted complexes and those of the reference complexes show the high quality of our predictions in an indirect way.

**Fig. 5:**
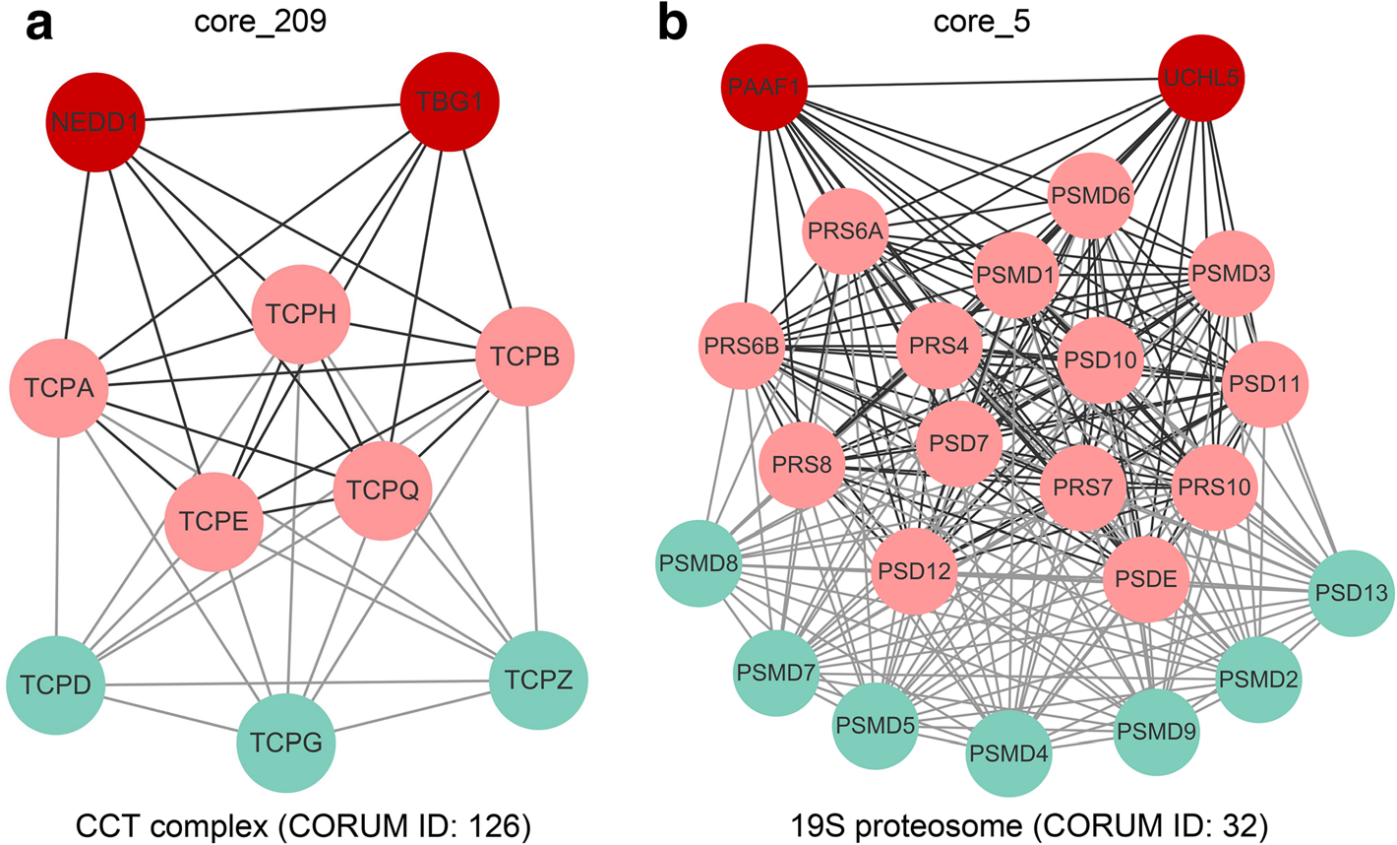
Two examples of potential WD40 protein-associated complexes. The nodes connected by dark grey lines belong to predicted complexes, whereas the nodes connected by light grey lines represent the reference complexes. Nodes in light red are shared by the predicted complex and reference complex. **a** the predicted complex, core_209, superimposed with the reference complex CCT complex; **b** the predicted complex, core_5, superimposed with reference complex 19S proteasome

Our complex prediction can provide valuable information for researchers studying WD40 proteins. For example, a predicted complex named “core_209” consists of seven proteins (Fig. 5a, see Additional file 3: Table S8), and among them, TCPA, TCPB, TCPE, TCPH, and TCPQ are subunits of the CCT chaperonin complex (CORUM complex ID: 126) [47]. The NEDD1 (WD40 protein) is not included in any complexes in the CORUM database, so the researchers studying on NEDD1 cannot obtain its complex information from CORUM but our predictions provided some. Furthermore, literature searching shows that NEDD1 was reported to localize at the centrosome and recruit γ-tubulin ring complex [48] through interacting with TBG1 (tubulin gamma-1 chain protein) [49]. And interestingly, one study has found that CCT can bind to unfolded γ-tubulin and promote its folding [50]. According to these studies, it is reasonable to propose that “core_209” might be a true complex in which the CCT bind to γ-tubulin to promote its folding, and then NEDD1 might recruit folded γ-tubulin ring complex (containing TBG1) to the centrosome.

Another example is “core_5” (Fig. 5b), which includes a WD40 protein (PAFF1) a protease (UCHL5), and many members of the 19S regulatory complex (CORUM complex ID: 32, PA700 complex) of the 26S proteasome. The database contains no information about whether it can interact with PAAF1 and UCHL5, but the predicted “core_5” suggests this possibility. Literature searching shows supporting evidences: The 19S regulatory complex recognizes poly-ubiquitinated proteins, recruits UCHL5 (a deubiquitinase) to removes the ubiquitins, and translocates them to the 20S core particle for degradation [51, 52]; PAAF1 interacts with the 19S regulatory complex, and destabilizes the association between the 19S complex and the 20S core [53], serving as a negative regulator of the 26S proteasome. Based on these clues, it is reasonable to propose that both UCHL5 and PAAF1 can bind the 19S regulatory complex to form a larger one. These examples demonstrate that our complex predictions based the network topology, in combination with literature mining, can provide informative clues to propose putative functions of WD40 proteins.

## Discussion

Network-based approaches have been applied to protein studies in recent years. During the last two decades, various methods and theories have been accumulated for biological network analyses concerning relationships between network features and proteins’ functions [20, 21, 23, 28, 54, 55]. Scardoni et al. discussed several topological centrality properties as well as their biological significances [56]. Highly connected proteins in a yeast interactome are found to tend to be essential [20], and the central located proteins are proposed more likely to be essential [21]. These established analysis strategies and the corresponding findings, in combination with the fast accumulated PPI data in online databases, make it possible to interrogate the distinct network characteristics of a specified protein set, such as the WD40 protein family.

The WD40 proteins are abundant in eukaryotes [57], and studies have suggested that they may expand into a large family in the evolutionary early stage of eukaryotes through the duplication events acted on the whole domain or protein [2]. In prokaryotes, a substantial proportion of WD40 proteins have been speculated with late origin through duplication events acted on the repeat level, although the total number of prokaryotic WD40s is much less than eukaryotic ones [58]. The reason why this family is prevalent in proteomes may stem from their structural and functional characteristics. According to the crystal structures of certain family members, the WD40 protein family is assumed to participate in protein-protein interactions and complex assembling, but there was no systematic confirmation. In this work, we have performed the first systematic and quantitative network analyses on human WD40 proteins. First, this work has shown that human WD40 protein family, as a whole, tends to be intramodular hubs and be located near the global center, leading to a better understanding concerning their roles in organizing the PPI networks. Second, we have provided quantitative measures for each WD40 protein concerning its network properties, such as degree and *k*-value, which can serve as clues to prioritize certain candidates for in-depth studies. On the other hand, these quantitative measures for each protein also provided information that could not otherwise be obtained from the overall tendency. For example, we found many non-hub WD40 proteins with very low connectivity, such as DC121, DC4 L1, DEND3, EMAL5, and TBL1Y.

Using only degrees, we cannot distinguish hub proteins located near the global center from those at the periphery, and the *k*-core decomposition can complement this deficiency. The *k*-core decomposition has demonstrated that WD40 proteins prefer to be located close to the global center of the PPI network, but not the local centers. The fact that the three WD40 proteins (MED16, GBLP, and COR1C) with top *k*-values are not the same as the three WD40s with top degrees, further shows the k-core decomposition has indeed added more information from another dimension.

In addition to static topological properties, the dynamic feature describing the average PCC between a hub and its interacting partners was also attempt, and it has revealed that WD40 hubs should tend to be intramodular, which quantitatively confirmed the previous inference that most WD40 proteins, if not all, should participate in various protein complexes. Inspired by this, we further predicted WD40-associated complexes from the topology of the human PPI network by using a simple clique-based method and three other well-known predictors. The comparison has revealed that, although the clique-base method may have a higher false positive rate, it can give out many more putative complexes with relatively high co-expression scores, which can serve as indicators of low false positive rates. The predicted novel complexes can also provide valuable clues to infer their detailed functions. In future work, one can seek to construct a negative set to evaluate the false positive rate directly.

We utilized two human PPI networks with different confidence levels. In all cases, the inferences drawn from these two networks are consistent, demonstrating that the overall conclusions in this work should be with enough robustness. In some cases, we can extrapolate the impacts stemming from different confidence levels: the tendency of WD40 proteins to be hubs can be higher when incorporating PPI data with low confidence, but many false positive complex predictions could be introduced. This also suggests that a more sophisticated clique-based method should be developed in the future, e.g., by integrating the confidence score of each PPI in the network and by training proper parameters for selecting informative interactions automatically.

## Conclusions

In summary, we have conducted the first systematic and quantitative network analyses on human WD40 proteins. By comparing with non-WD40 proteins on several static topological properties and a dynamic feature by integrating co-expression data, our work demonstrated that the WD40 family tend to be intramodular hubs and be located near the global center of the whole network, providing clues about their roles in organizing the PPI network. In addition, these findings have quantitatively confirmed that the previous structure-based inference that the WD40 protein family may often act as scaffolds to assemble complexes. Finally, we have effectively predicted the WD40 protein-associated complexes by using a clique-base method. The quantitative features analyzed in this work and the predicted complexes, can serve as clues for inferring putative functions and prioritizing candidates for further studies.

## Methods

### Protein-protein interaction data set and network construction

The human PPI data set was downloaded from the HIPPIE database [26, 27] (v2.0, release 2016–05-24), which presents one of the most comprehensive human PPI data set. It has integrated experimentally detected PPIs extracted from MINT [16], MIPS [17], HPRD [18], IntAct [59], BioGRID [60], DIP [61] and BIND [62], and has also implemented a confidence scoring system weighting the amount and quality of the evidences for each interaction. The larger the score (ranging from 0 to 1) is, the higher the confidence is. After downloading the data set (273,927 interactions at the moment when accessed), we further cleaned it by removing PPIs lacking the UniProt ID [34] or describing self-interactions, and the repetitive interactions were also merged. This process of data cleaning resulted in the ALL-PPI data set. Based on it, we curated the HC-PPI (high confidence PPI) data sets by keeping only the PPIs whose confidence scores are at least 0.72, the third quartile of all the scores, which was also suggested for filtering out potential false positive interactions by the authors of HIPPIE. In practice, as the confidence scores take values from 65 different ones, and more than 25,000 interactions have the score of 0.72, the percentage of interactions in HC-PPI to those in ALL-PPI was greater than 25%. A list of 262 human WD40 proteins was retrieved from previous work [2], and was adopted to label the WD40 proteins in ALL-PPI and HC-PPI. All other proteins were treated as non-WD40s.

A PPI network is defined as a graph, where nodes and edges represent proteins and their interactions, respectively. In the network, there may be isolated components without any edge connecting them, and the largest one is referred to as the main component. We adopted Cytoscape [63] to construct the PPI networks for the ALL-PPI and HC-PPI, and the topological parameters were calculated by NetworkAnalyzer [63].

### Centralities and other properties comparison between WD40 and non-WD40 proteins

Centralities are basic network properties to characterize each node or edge with respect to their positions within the network. The comparison of centrality between WD40 and non-WD40 proteins was mainly conducted by utilizing the degree measure, which is the most intuitive. Other measures including betweenness, closeness, and clustering coefficient were also attempted.

The degree of a node in network is the number of its direct links with other nodes. In PPI network, a highly connected protein (say, degree greater than 5) is defined as a ‘hub’, as described in previous publications [23, 24]. We mainly adopted this cutoff to define hubs, and the cutoff of 10 and 15 were also used for extended comparisons. The ratios of hubs to non-hubs were calculated for WD40 and non-WD40 proteins, respectively. The odds ratio (OR) was defined by dividing this ratio in WD40 proteins to that in non-WD40s. The χ^2^ test was adopted to measure the statistical significance that the odds ratio differs from 1.

Betweenness centrality of a node in network reflects the amount of control that this node exerts over interactions of other nodes in the network [64]. The betweenness of node *n* is calculated as follows:

1 $$ {C}_b(n)={\sum}_{s\ne n\ne t}\left({\sigma}_{st}(n)/{\sigma}_{st}\right), $$

where *s* and *t* are nodes in the network different from node *n*, *σ*_*st*_ denotes the number of shortest paths from *s* and *t*, and *σ*_*st*_(*n*) is the number of shortest paths from *s* to *t* that node *n* lies on. In NetworkAnalyzer, the betweenness value for each node *n* is further normalized by dividing by the number of node pairs excluding node *n*.

Closeness centrality measures how fast information spreads from a given node to other reachable nodes in the network. Closeness centrality of node *n* is defined as the reciprocal of the average shortest path length [65], and it can be calculated as follows:

2 $$ {C}_c(n)=1/ avg\left(L\left(m,n\right)\right), $$

where *L*(*m*, *n*) is the length of shortest path between node *n* and *m*, and *m* denotes any other nodes that are reachable to node *n*.

Stress centrality of a node *n* is calculated by the number of shortest paths passing through node *n*. A high stress centrality means traversed by a lot of shortest paths [66].

In PPI networks, the clustering coefficient of a node *n* is defined as follows:

3 $$ {C}_n=2{e}_n/\left({k}_n\left({k}_n-1\right)\right), $$

where *k*_*n*_ denotes the number of neighbors of node *n*, *e*_*n*_ is the number of connected pairs between all neighbors. This property measures the trend of forming a cluster by node *n* and its neighbors [28].

All the network properties described above were calculated for each protein in both HC-PPI network and ALL-PPI network through NetworkAnalyzer [63]. Direct comparisons of them between WD40 and non-WD40 proteins were performed by using single-tailed Mann-Whitney U test. The fold changes measuring the ratio of median degree of WD40 proteins to that of non-WD40 were also calculated.

Expression patterns of top high- and low-degree WD40 proteins were retrieved directly from a previous study [2]. They were based on the RNA-seq data set in the Human Protein Atlas project [35], which was further utilized in the following sections in this study. Proteins expressed in all 27 tissues with FPKM > 10 are defined as “High in all tissues”, and those expressed in most (but not all) tissues with FPKM > 10 are defined as “High in many tissues”. Proteins expressed in one tissue with FPKM 5 or more times greater than in all other tissues are defined as “Tissue-specific”, and those demonstrate expression preference in specific tissues, but fold changes were less than 5, are named as “Tissue-preferential”.

### *k*-core decomposition of PPI network

The *k*-core decomposition [67] of a PPI network was carried out by iteratively removing all nodes with degree less than *k* until all the remaining nodes have degrees of at least *k*. The remaining part is named as the *k*-core subnetwork accordingly. When the *k* increases stepwise from 1, the locations of the remaining nodes go from the periphery to the center of the whole network (Additional file 1: Figure S9a). This decomposition process splits the network into different layers from outside to inside, where the layer *k* contains proteins in the *k*-core subnetwork but excluding those in the (*k + 1*)-core subnetwork. Each protein in layer *k* can then be assigned the value of *k* (i.e., the *k*-value) to describe its layer location. The larger the *k*-value is, the closer to the center of the whole network (i.e., the global center) the node is. Nodes with high degrees but low *k*-values are hubs located at the periphery, and are named as local centers (Additional file 1: Figure S9b) [21].

The *k*-core decomposition described above was applied to the HC-PPI and ALL-PPI network respectively. For comparison, the median *k*-values for WD40 and non-WD40 proteins in each network, and the percentage of WD40s in each *k*-core subnetwork were calculated respectively. A fold change was measured by the ratio of median *k*-value of WD40 proteins to that of non-WD40s.

The list of human essential genes was retrieved from a previous study [36]. It contains 1299 genes which were integrated from four distinct sources. Evolutionary conservation analysis of WD40 proteins near the global center was performed by checking their orthologs in other model eukaryotes in the Inparanoid database [68].

### Analysis of the intramodular preference for WD40 hubs

Using the gene expression data from a series of different tissues, one can calculate the Pearson’s correlation coefficient (PCC) to quantify the extent to which a pair of interacting proteins were co-expressed. Here, the expression data of a gene was represented as a vector consisting of the same number of components as the number of different tissues. According to previous studies in yeast and human interactome [23, 24], the average of all PCCs between a hub and its interacting partners can be adopted to identify whether interactions of this hub are context-specific (low average PCC) or constitutive (high average PCC), and this hub is referred to as intermodular or intramodular accordingly.

The distribution of the average PCCs of the WD40 hubs was compared with that of non-WD40 hubs. As a control, we also generated a random distribution of the average PCCs of all hub proteins. In brief, the associations between the expression vectors and proteins were shuffled, and then the average PCC for each hub protein was re-calculated to generate this random distribution. The same analyses were carried out on HC-PPI and ALL-PPI network, respectively. For each one, both RNA-level and protein-level expression data set were considered independently.

For the RNA-level expression, we used the RNA-seq data set in the Human Protein Atlas project (ArrayExpress ID: E-MTAB-1733) [35]. This data set contains the RNA expression levels in terms of the FPKM values for 20,050 protein-coding genes in 27 different tissues from 95 samples. In each tissue, the FPKM values of a gene from different samples were averaged to represent its expression level, and its expression values from different tissues constitute the expression vector. The expression vectors of a protein pair were used for the PCC calculation. The UniProt ID mapping tool [34] and bioDBnet [69] were adopted to map the IDs in the RNA-seq data set and the protein IDs in the PPI networks. After ID mapping and deletion of ambiguities, 15,358 and 10,751 proteins in the ALL-PPI and the HC-PPI network were assigned expression data, respectively.

For the protein-level expression, we utilized the data from Human Proteome Map [70], which contains expression information for more than 30,000 proteins in 30 human tissues. After ID mapping and deletion of ambiguities, 13,764 and 10,003 proteins in the ALL-PPI and the HC-PPI network were assigned expression data, respectively.

### Complex predictions

In the clique-based method, we took three simple steps to mine WD40-associated complexes in the PPI network. First, we extracted a subnetwork that only contains WD40 proteins and their directly connected neighbors (first-order neighbors). Second, all maximal cliques were identified based on the algorithm developed by Bron et al. [71]. Third, maximal cliques with size greater than 2 and containing at least one human WD40 proteins were chosen to be potential WD40-associated complexes.

Since some cliques generated above may overlap with others, two cliques can be merged according to a specified merging parameter that measures the proportion of overlapped protein number to that in the smaller clique. To determine to what extent the overlapped cliques should be merged together, we tried several merging parameters (50%, 60%, 70%, 80%, 90%, and 100%), resulting in a series of predicted complex sets (namely M05, M06, M07, M08, M09, and M10, respectively). For example, predicted complex set M05 is obtained from iteratively merging cliques that share 50% nodes, and M10 means no merging at all.

The reference complex data set contains all 234 experimentally identified human WD40-associated protein complexes extracted from the CORUM database [43], where 90 human WD40 proteins were involved. The overlap score ω [44], which was used to determine whether a predicted complex “matches” one of the complexes in the reference set, was defined as:

4 $$ \upomega \kern0.5em =\kern0.5em \frac{{\left|A\kern0.5em \cap \kern0.5em B\right|}^2}{\left|A\right|\ast \left|B\right|}, $$

where A and B represent the complex A and complex B, and |A| and |B| represent the number of proteins in them, respectively. We tried a series of scores of ω comprehensively, from 0.0 to 1.0, to evaluate our complex prediction results from different merging parameters mentioned above. This evaluation could help to choose a proper merging parameter.

For comparison, other methods including MCODE [44], ClusterOne [45], and MCL [46] were also attempted. These methods took two steps to predict WD40 protein-associated complexes from the main component of the PPI network. First, they detected all so-called “modules” in the main component of the PPI network; Second, “modules” with size of at least 3 and containing at least one WD40 protein were kept as potential complexes. According to the recommendation of the original literatures, default settings were chosen for both ClusterOne and MCODE, whereas three different values (1.5, 2.0, and 4.0) were used to control the granularity for MCL, respectively.

To compare different complex prediction methods, we calculated the numbers of matched reference complexes by predicted complexes from each method, and further utilized a measure called maximal matching ratio (MMR) [45], which is a well-known index that evaluates the overall level of overlap between the matched reference complexes and the predicted complexes that matching these reference complexes.

### Co-expression scores of predicted complex set, randomized protein set, and reference set

Co-expression score of a complex (or a protein set) was calculated through two steps. In the first step, we calculated the PCCs between any two proteins within the complex. In the second step, the mean of these PCCs was computed as the co-expression score of this complex.

The randomized data set, which was used for comparison, was generated by random sampling from the HC-PPI network. It contained the same number of “decoy complexes” as that in the predicted set. The numbers of member proteins were also the same as those in the predicted set, but they were randomly chosen from the main component of HC-PPI network.

The expression data sets used here were the same as described in the calculation of average PCCs for hub proteins. The co-expression scores of the predicted complexes, the reference complexes, and the “decoy complexes” were all independently calculated based on the protein-level expression and the RNA-level expression data.

## Additional files

Additional file 1: **Figure S1.** Workflow of this study. **Figure S2.** Overview of the ALL-PPI network. **Figure S3.** Degree distributions of nodes in two networks. **Figure S4.** Percentage of WD40 proteins in each *k*-core subnetwork during the decomposition of ALL-PPI network. **Figure S5.** Distributions of average PCCs of WD40 hubs, non-WD40 hubs, and randomized data in ALL-PPI network. **Figure S6.** The number of reference complexes matched with predicted complexes obtained by different methods. **Figure S7.** The number of reference complexes matched with predicted complexes obtained from ALL-PPI network under different **ω**. **Figure S8.** Number of complexes in reference set matched with predicted complexes obtained from different PPI networks. **Figure S9. ***k*-core decomposition and localization of hubs in PPI network. **Table S2.** Counts of hubs in WD40 and non-WD40 proteins in ALL-PPI network. **Table S3.** Counts of hubs in WD40 and non-WD40 under different definitions. **Table S4.** Comparisons of centralities between WD40 and non-WD40 in two networks. **Table S5.** WD40 proteins in different layers obtained by *k*-core decomposition of ALL-PPI network. **Table S6.** Orthologs of MED16, GBLP, and CORO1C in model organisms. **Table S7.** Medians of average PCCs of WD40 hubs and non-WD40 hubs in two networks. **Table S9.** Statistics of complex predictions  based on HC-PPI under different parameter settings. **Table S11.** Number of predicted complexes under different methods, and the matched numbers of reference complexes under different ω. **Table S12.** Comparisons of MMR for different prediction methods with ω> = 0.2. **Table S13.** Statistics of complex prediction obtained from ALL-PPI under different parameter settings. **Table S14.** Medians of co-expression scores for different complex sets under different expression dataset. (PDF 1014 kb)

Additional file 2: **Table S1.** ALL-PPI (including HC-PPI) interactions annotated with confidence scores and PCCs. (XLSX 9754 kb)

Additional file 3: **Table S8.** Maximal cliques derived from HC_PPI network. (XLSX 103 kb)

Additional file 4: **Table S10.** Reference human WD40 complexes derived from the CORUM database. (XLSX 27 kb)

## Abbreviations

ALL-PPI: all PPI data obtained from the HIPPIE database after data cleaning
HC-PPI: high confidence PPI data from ALL-PPI with confidence score greater than the third quantile (0.72)
HIPPIE: Human integrated protein-protein interaction reference
OR: odds ratio
PCC: Pearson’s correlation coefficient
PPI: protein-protein interaction
