# Supplementary material for: PPI network analyses of human WD40 protein family systematically reveal their tendency to assemble complexes and facilitate the complex predictions
Source: BMC Syst Biol. 2018 Apr 24;12(Suppl 4):41. doi: 10.1186/s12918-018-0567-9 (PMC5998875; doi:10.1186/s12918-018-0567-9)
Supplement: Supplementary file 1 — Figure S1. Workflow of this study. Figure S2. Overview of the ALL-PPI network. Figure S3. Degree distributions of nodes in two networks. Figure S4. Percentage of WD40 proteins in each k-core subnetwork during the decomposition of ALL-PPI network. Figure S5. Distributions of average PCCs of WD40 hubs, non-WD40 hubs, and randomized data in ALL-PPI network. Figure S6. The number of reference complexes matched with predicted complexes obtained by different methods. Figure S7. The number of reference complexes matched with predicted complexes obtained from ALL-PPI network under different ω. Figure S8. Number of complexes in reference set matched with predicted complexes obtained from different PPI networks. Figure S9. k-core decomposition and localization of hubs in PPI network. Table S2. Counts of hubs in WD40 and non-WD40 proteins in ALL-PPI network. Table S3. Counts of hubs in WD40 and non-WD40 under different definitions. Table S4. Comparisons of centralities between WD40 and non-WD40 in two networks. Table S5. WD40 proteins in different layers obtained by k-core decomposition of ALL-PPI network. Table S6. Orthologs of MED16, GBLP, and CORO1C in model organisms. Table S7. Medians of average PCCs of WD40 hubs and non-WD40 hubs in two networks. Table S9. Statistics of complex predictions based on HC-PPI under different parameter settings. Table S11. Number of predicted complexes under different methods, and the matched numbers of reference complexes under different ω. Table S12. Comparisons of MMR for different prediction methods with ω> = 0.2. Table S13. Statistics of complex prediction obtained from ALL-PPI under different parameter settings. Table S14. Medians of co-expression scores for different complex sets under different expression dataset. (PDF 1014 kb) [file 12918_2018_567_MOESM1_ESM.pdf]

Supplementary Figures:

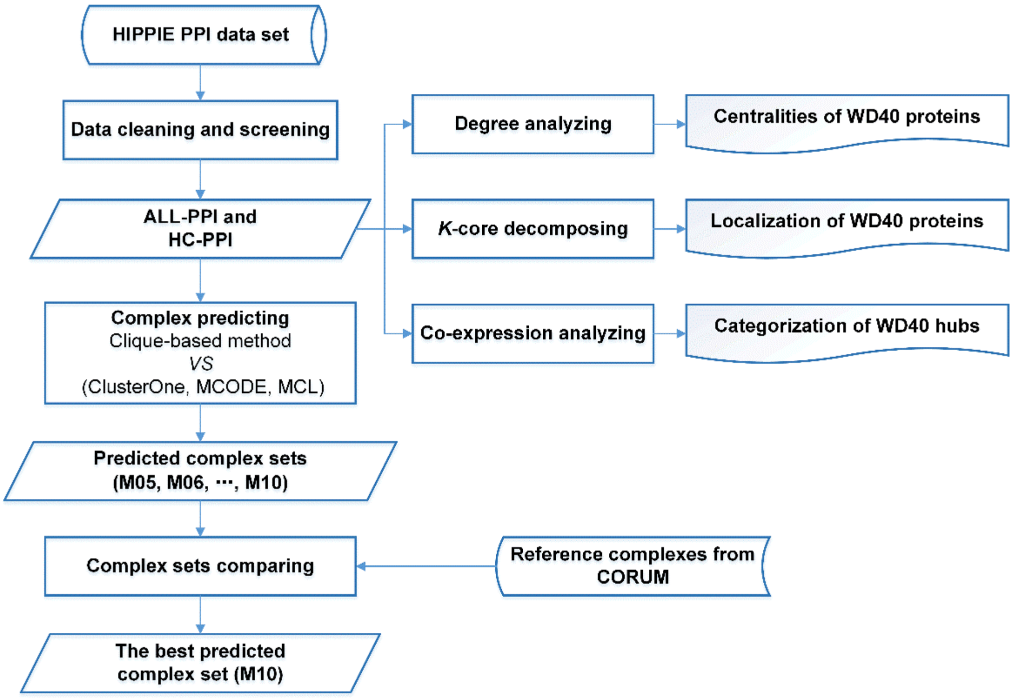

Figure S1. Workflow of network analysis and complex prediction of WD40 proteins.

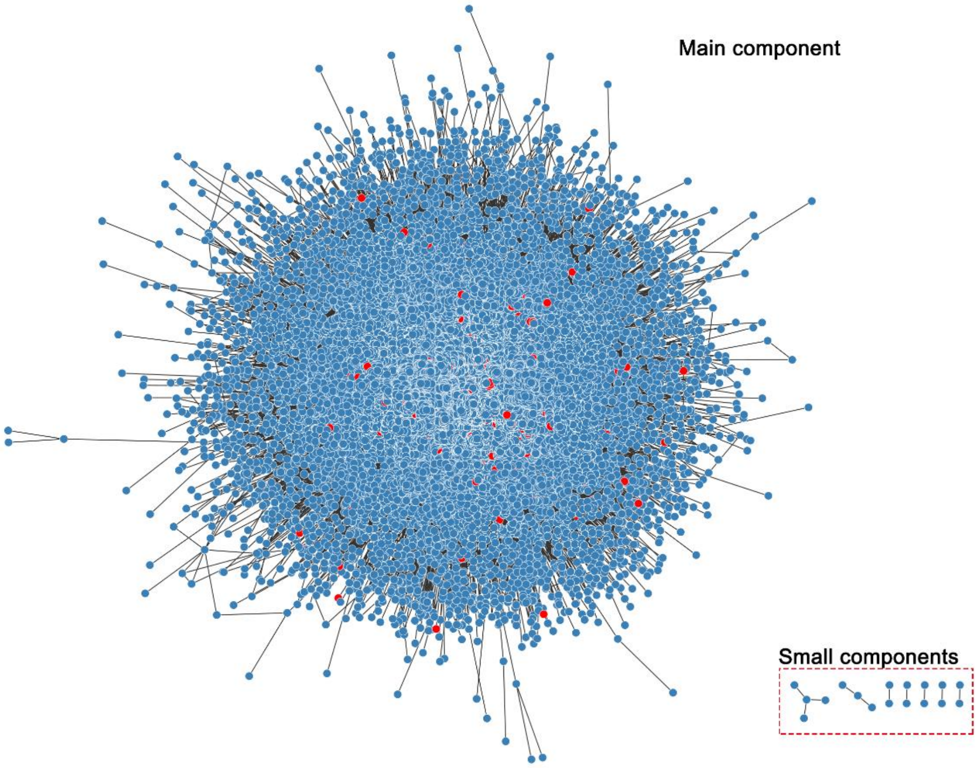

Figure S2. Overview of ALL-PPI network. The visualization was carried out by Cytoscape [1] with “Prefuse Force Directed Layout”. It consists of a large main component and seven small components (red box). Nodes filled with red are WD40 proteins.

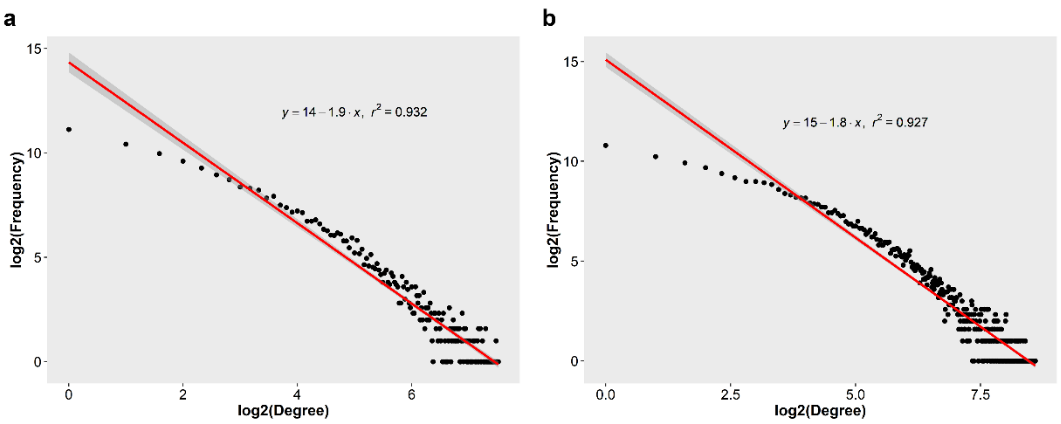

Figure S3. Log transformed degree distributions of nodes in HC-PPI network and ALL-PPI network. The red lines are fitted by simple linear regression model, and the R^2 are 0.932 (p<2.2e-16) and 0.927 (p<2.2e-16) for HC-PPI network and ALL-PPI, respectively. a, the degree distribution of HC-PPI network; b, the degree distribution of ALL-PPI network.

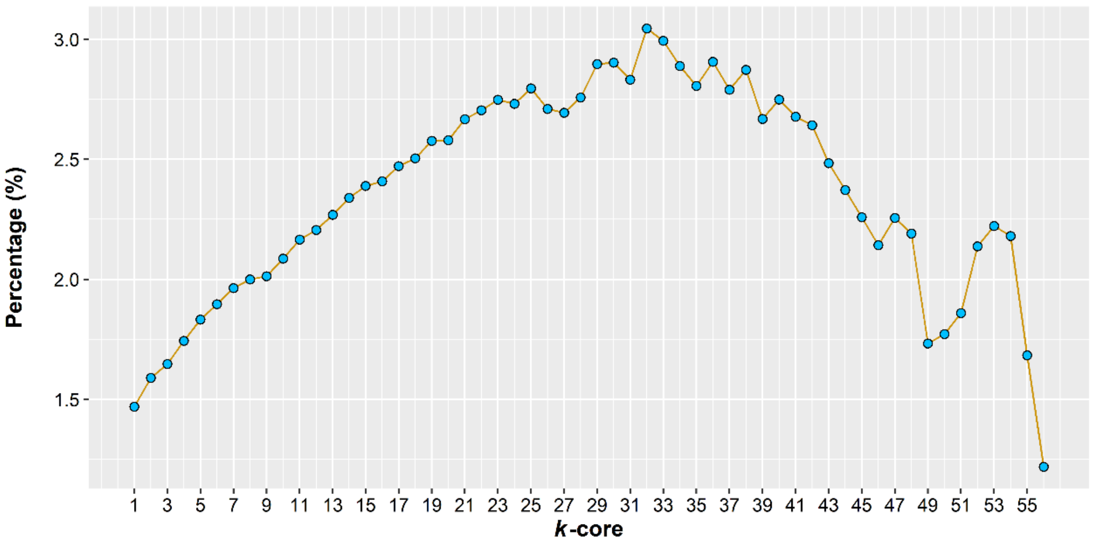

Figure S4. Percentage of WD40 proteins in each *k*-core subnetwork during the decomposition of ALL-PPI network.

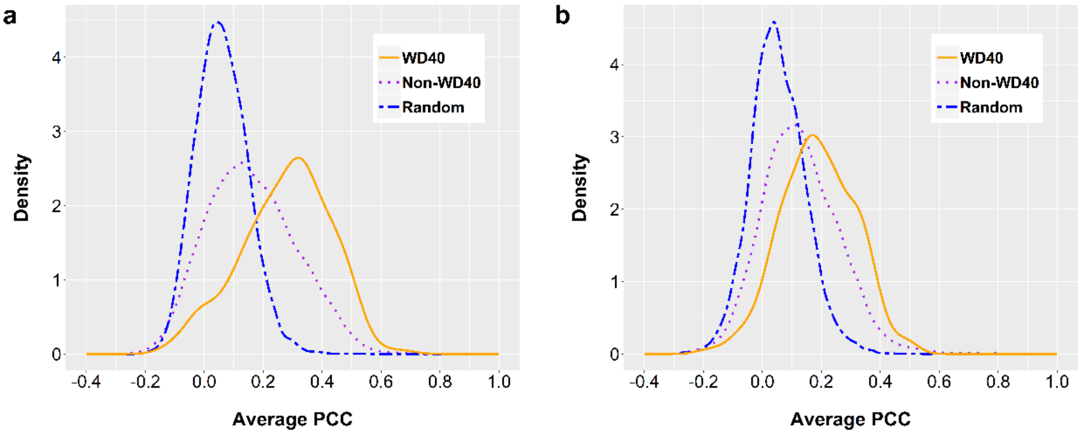

Figure S5. Distributions of average PCCs of WD40 hubs, non-WD40 hubs, and randomized data in ALL-PPI network. The meanings of the lines are the same as in Figure 2.

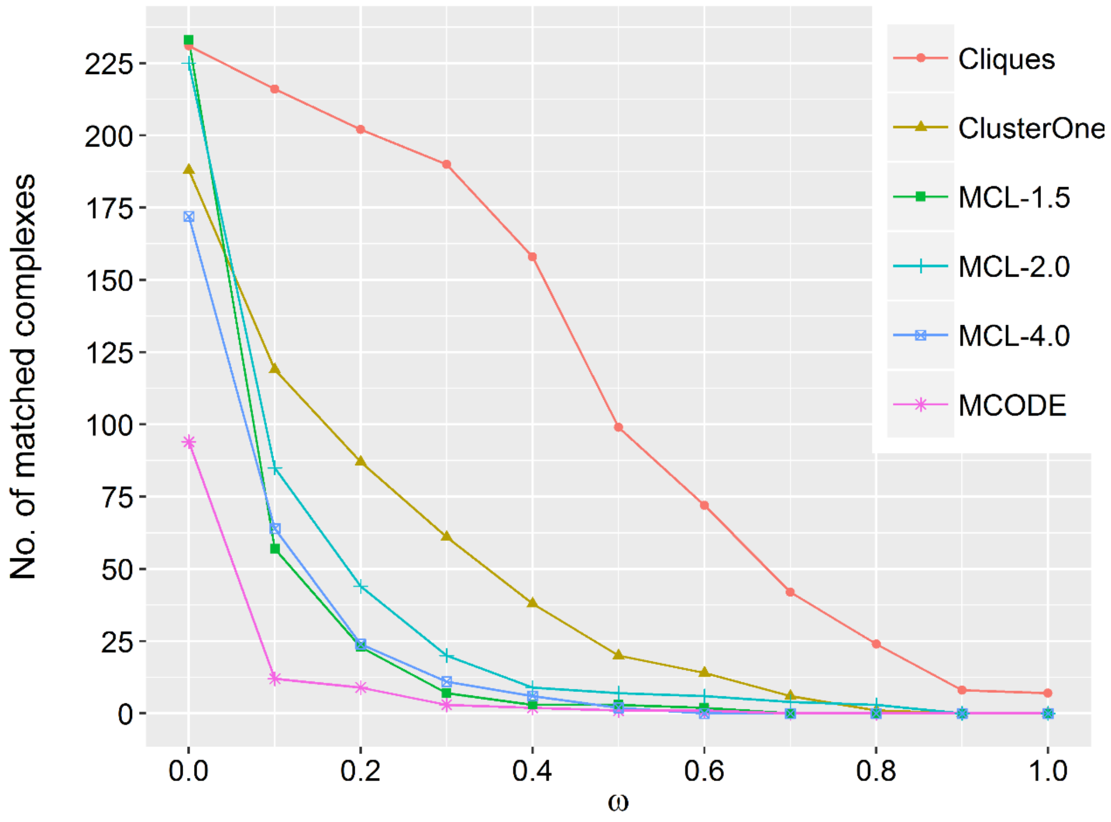

Figure S6. The number of reference complexes matched with predicted complexes obtained by different methods under different ω scores. The line labelled as “Cliques” represents M10 (see Methods) complex set from clique-based method; MCL-1.5, MCL-2.0, and MCL-4.0 represent complex sets obtained by MCL method under three different values of the parameter describing granularity (see Methods).

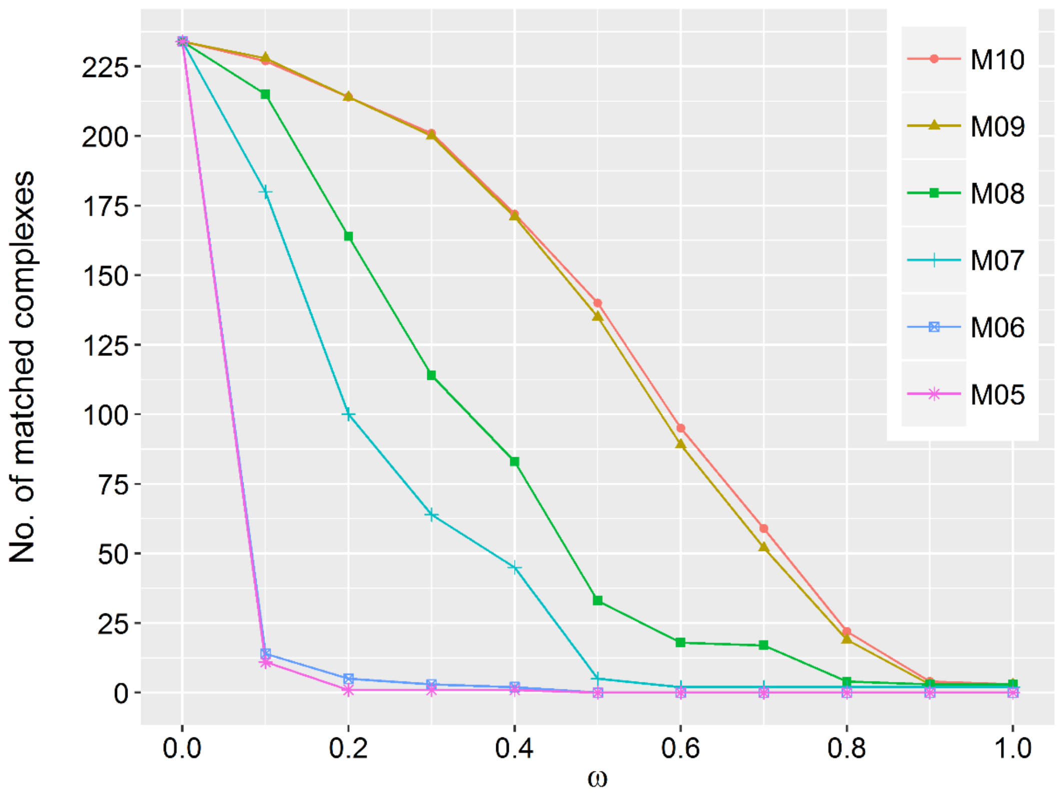

Figure S7. The number of reference complexes matched with predicted complexes obtained from ALL-PPI network under different ω scores. M05 to M10 here are the same with those in Figure 3.

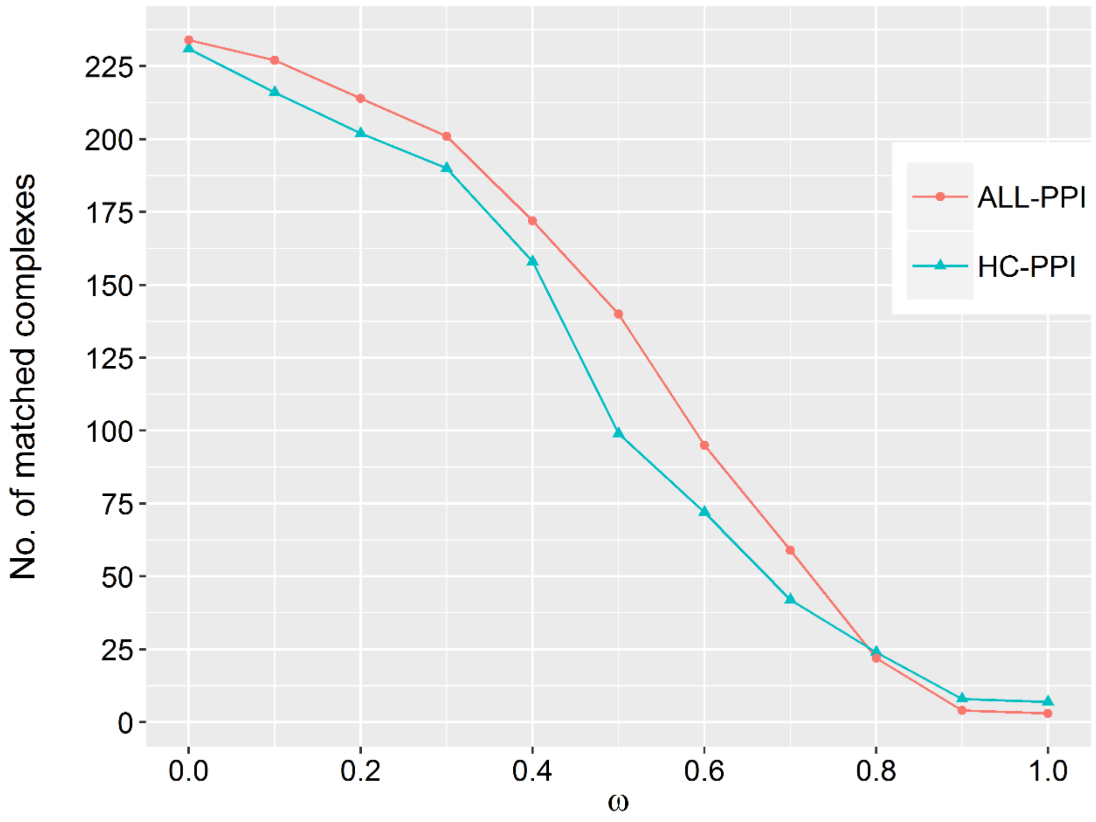

Figure S8. Number of complexes in reference set matched with predicted complexes obtained from different PPI networks.

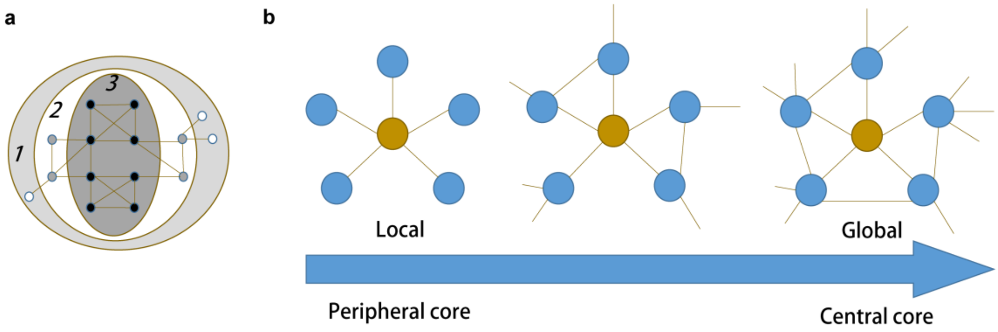

Figure S9. Schematic representation of *k*-core decomposition and localization of hubs in PPI network.

a, A network that has been decomposed into three core subnetworks, namely 1-core (the outermost ellipse), 2-core (the middle ellipse), and 3-core (the innermost ellipse). Layer 1 includes three nodes filled with light grey, Layer 2 contains four nodes filled with white, and the eight nodes filled with black are located at Layer 3; b, the topology of hubs at different locations from peripheral core towards central core.

Supplementary Tables:

Table S2 Counts of hubs in WD40 and non-WD40 proteins in ALL-PPI network

	WD40	Non-WD40	Total
**Hub**	205	10578	10783
**Non-hub**	37	5406	5443
**Total**	242	15984	16226

The odds ratio (OR) for WD40 to be hubs here is 2.83 and χ^2 independent test for this gives a *p*-value of 2.077e-9, suggesting WD40 proteins are significantly more likely to be hubs.

Table S3 Counts of hubs in WD40 and non-WD40 under different definitions and networks

Counts of hub (degree > 10)						
	HC-PPI			ALL-PPI		
	WD40	Non-WD40	Total	WD40	Non-WD40	Total
**Hub**	89	3174	3263	178	8066	8244
**Non-hub**	114	7731	7845	64	7918	7982
**Total**	203	10905	11108	242	15984	16226
	OR = 1.90, *p* = 7.14e-6			OR = 2.73, *p* = 1.59e-12		
**Counts of hub (degree > 15)**						
**Hub**	64	2238	2302	153	6470	6623
**Non-hub**	139	8667	8806	89	9514	9603
**Total**	203	10905	11108	242	15984	16226
	OR = 1.78, *p* = 1.8e-4			OR = 2.53, *p* = 1.45e-12		

OR: odds ratio. The statistic test used is χ^2 independent test.

Table S4 Comparisons of centralities between WD40 and non-WD40 in HC-PPI and ALL-PPI network

	HC-PPI			ALL-PPI		
	WD40	Non-WD40	*p*	WD40	Non-WD40	*p*
**Degree**	9	5	2.19e-8	24	11	8.09e-15
**Betweenness**	3.15e-05	1.39e-05	9.59e-4	2.25e-05	8.38e-06	2.97e-7
**Closeness**	0.281	0.269	4.16e-6	0.344	0.326	6.48e-18
**Stress**	63700	28430	2.96e-4	254700	82570	2.94e-9
**Clustering coefficient**	0.129	0.036	1.10e-9	0.124	0.073	4.25e-11

The value under WD40 and non-WD40 are median values of degree, betweenness, closeness, stress, and clustering coefficient, respectively. The last column is P-values obtained by single tailed Mann-Whitney U test.

Table S5 WD40 proteins in different layers obtained by *k*-core decomposition of ALL-PPI network.

K-layer	No.WD40	No.non-WD40	WD40 protein
56	3	240	EED, 2ABB, PAN2
55	2	47	GBLP, FBW1B
54	2	20	FBW1A, DDB1
53	1	37	PRP19
52	1	59	RBBP4
51	0	63	-
50	0	24	-
49	1	67	CDC20
48	4	54	EIF3I, RBBP7, WDR5, EIF3B
47	1	24	MEP50
46	1	80	COR1C
45	2	46	BUB3, DCAF7
44	2	42	FBXW7, STRAP
43	3	77	GBB2, RAE1L, COPA
42	3	52	2ABA, NEDD1, GEMI5
41	2	58	VPRBP, PRP4
40	4	110	DDB2, RPTOR, SNR40, RFWD2
39	1	71	FZR
38	5	76	GBB1, WDR82, ARC1B, WDR1, SEC13
37	2	107	RBBP5, TLE1
36	6	137	TBL1R, EDC4, STRN4, DTL, STRN, MED16
35	1	90	WRP73
34	5	115	WDR48, SEH1, WDR76, FBXW4, STRN3
33	7	157	LIS1, WDR18, WDR83, WDR26, COPB2, CIAO1, WDR33
32	6	152	TBL1X, PLRG1, DC1I2, 2ABD, PWP1, WDR61
31	2	227	A16L1, DCAF8
30	6	137	EIF2A, PAAF1, TF3C2, CAF1B, COR1B, FBXW8
29	5	168	ERCC8, KI21A, HIRA, THOC6, PRP17
28	1	170	DC1I1
27	4	208	WDR62, WIPI2, BOP1, LRWD1
26	5	157	2ABG, NUP43, MABP1, TBL3, WDHD1
25	10	238	CIR1A, LST8, PLAP, WDR6, TAF5, WDR4, FBXW5, ARC1A, FAN, WDR36
24	3	186	SMU1, SC31A, TAF5L
23	7	218	WDR12, GBB4, PALB2, TLE3, COR1A, LYST, AAMP
22	4	204	WDR92, UTP18, ELP2, PWP2
21	7	306	TRAF7, KI21B, GBB5, TSSC1, HERC1, PHIP, WAP53
20	3	261	KCTD3, WDR20, APAF
19	7	264	CORO7, SHKB1, DCA11, AAAS, DCAF5, TBL2, COR2A
18	3	259	PK1IP, PI3R4, CSTF1

17	6	300	NUP37, WDR3, WDR43, FBXW2, PEX7, UTP15
16	5	344	NLE1, WDTC1, WDR44, CD20B, DCA10
15	9	406	WDR90, WDR37, GBB3, THOC3, SCAP, WDR74, BRWD1, WDFY2, U3IP2
14	6	379	TLE2, LRBA, WIPI1, DCA13, WDR34, WDR75
13	4	378	MIO, DCAF4, WSB1, WDR91
12	4	378	HPS5, WDR46, SC31B, WDR73
11	7	449	WDR59, STXB5, WIPI3, EMAL4, IF140, WDR81, WDR24
10	4	486	WDR35, IF172, WDR5B, NBEA
9	4	501	DMXL2, GNB1L, TEP1, NOL10
8	11	587	WIPI4, KTNB1, WSB2, IF122, NBEL1, WDR55, EMAL1, BRWD3, WDR47, AHI1, A16L2
7	8	570	POC1B, EMAL2, WDR7, EMAL3, WDR70, WDFY1, WDR25, WDR89
6	5	611	DCA12, NBEL2, DMXL1, DC4L2, WDR54
5	6	693	TCPR2, FBXW9, COR2B, POC1A, WDR41, WDR16
4	5	860	DEND3, PREB, WDR53, IFT80, WDR60
3	5	1023	DC4L1, CORO6, STB5L, DC122, WDR19
2	12	1219	WDR27, WDR65, DNAI1, SPG16, PPWD1, WDR66, TBL1Y, FBW12, DNAI2, EMAL5, WDR13, FBW10
1	9	1792	TLE4, DC121, WDR88, WDR52, WSDU1, RIC1, K1875, WDR63, TLE6

Table S6 Orthologs of MED16, GBLP, and CORO1C in model organisms.

	Mouse	Chiken	Fly	Frog	Zebrafish	Nematoda	Arabidopsis	Yeast
MED16	+	+	+	+	+	-	-	-
GBLP	+	+	+	+	+	+	+	+
CORO1C	+	+	+	+	+	+	-	-

Orthologs for above genes were obtained from Inparanoid database [2]. “+” means an ortholog exists in the corresponding organism, “-“ means the opposite.

Table S7. Medians of average PCCs of WD40 hubs and non-WD40 hubs in HC-PPI and ALL-PPI networks

		HC-PPI	ALL-PPI
Protein-level	WD40 hub	0.343 (118)	0.296 (199)
	Non-WD40 hub	0.217 (4693)	0.150 (9574)
	P-value	1.7e-10	8.0e-25
RNA-level	WD40 hub	0.221 (122)	0.180 (200)
	Non-WD40 hub	0.171 (4817)	0.120 (9886)
	P-value	1.6e-4	7.0e-12

Numbers in parenthesis represent the counts of WD40 hub and non-WD40 hub.

Table S9 Statistics of predicted complexes based on HC-PPI under different merging parameters, and the numbers of matched reference complexes under different ω score

	M10	M09	M08	M07	M06	M05
No. predicted complexes	1674	1606	1120	730	92	80
Range of complex size	3~16	3~21	3~76	3~326	3~846	3~1000
No. WD40 involved	150	150	150	150	150	150
ω > 0.0	231	231	231	231	231	231
ω >= 0.1	216	216	209	196	43	37
ω >= 0.2	202	202	180	132	20	15
ω >= 0.3	190	189	138	90	18	12
ω >= 0.4	158	158	105	67	13	9
ω >= 0.5	99	99	47	22	9	6
ω >= 0.6	72	70	31	18	8	6
ω >= 0.7	42	41	19	10	2	2
ω >= 0.8	24	21	8	4	1	1
ω >= 0.9	8	7	3	2	1	1
ω >= 1.0	7	7	3	2	1	1

Table S11 Number of predicted complexes under different methods, and the matched numbers of reference complexes under different ω score.

	Clique-based	ClusterOne	MCODE	MCL-1.5	MCL-2.0	MCL-4.0
No. predicted complexes	1674	76	8	96	116	90
Range of complex size	3~16	3~37	4~69	3~1197	3~157	3~32
No. WD40 involved	150	85	14	195	175	118
ω > 0.0	231	188	94	233	225	172
ω >= 0.1	216	119	12	57	85	64
ω >= 0.2	202	87	9	23	44	24
ω >= 0.3	190	61	3	7	20	11
ω >= 0.4	158	38	2	3	9	6
ω >= 0.5	99	20	1	3	7	2
ω >= 0.6	72	14	1	2	6	0
ω >= 0.7	42	6	0	0	4	0
ω >= 0.8	24	1	0	0	3	0
ω >= 0.9	8	0	0	0	0	0
ω >= 1.0	7	0	0	0	0	0

Table S12. Comparisons of MMR for different prediction methods with ω >= 0.2.

Clique-based	MCODE	ClusterOne	MCL-1.5	MCL-2.0	MCL-4.0
0.48	0.41	0.47	0.31	0.41	0.35

Table S13. Statistics of predicted complexes obtained from ALL-PPI under different merging parameters, and the number of matched reference complexes under different ω scores.

	M10	M09	M08	M07	M06	M05
No. predicted complexes	23416	22743	9023	3541	70	59
Range of complex sizes	3~22	3~52	3~1715	3~2416	3~3263	3~3291
No. WD40 involved	215	215	215	215	215	215
$\omega > 0.0$	234	234	234	234	234	234
$\omega >= 0.1$	227	228	215	180	14	11
$\omega >= 0.2$	214	214	164	100	5	1
$\omega >= 0.3$	201	200	114	64	3	1
$\omega >= 0.4$	172	171	83	45	2	1
$\omega >= 0.5$	140	135	33	5	0	0
$\omega >= 0.6$	95	89	18	2	0	0
$\omega >= 0.7$	59	52	17	2	0	0
$\omega >= 0.8$	22	19	4	2	0	0
$\omega >= 0.9$	4	3	3	2	0	0
$\omega >= 1.0$	3	3	3	2	0	0

Table S14 Medians of co-expression scores for predicted complexes, reference complexes, and decoy complexes with both protein-level and RNA-level expression data

	Predicted set	Reference set	Decoy set
Protein-level expression	0.488	0.532	0.225
RNA-level expression	0.358	0.374	0.237

References

1. Shannon P, Markiel A, Ozier O, Baliga NS, Wang JT, Ramage D, Amin N, Schwikowski B, Ideker T: **Cytoscape: a software environment for integrated models of biomolecular interaction networks**. *Genome Res* 2003, **13**(11):2498-2504.
2. Sonnhammer EL, Ostlund G: **InParanoid 8: orthology analysis between 273 proteomes, mostly eukaryotic**. *Nucleic Acids Res* 2015, **43**(Database issue):D234-239.
